# Clathrin-independent endocytosis: an increasing degree of complexity

**DOI:** 10.1007/s00418-018-1678-5

**Published:** 2018-05-17

**Authors:** Kirsten Sandvig, Simona Kavaliauskiene, Tore Skotland

**Affiliations:** 1 Department of Molecular Cell Biology, Institute for Cancer Research, The Norwegian Radium Hospital, Oslo University Hospital, Montebello, 0379 Oslo, Norway; 2 Department of Molecular Biosciences, University of Oslo, 0316 Oslo, Norway

**Keywords:** Endocytosis, Clathrin, Caveolin, Caveolae, Endophilin, Rho proteins

## Abstract

This article aims at providing an update on the complexity of clathrin-independent endocytosis. It is now almost 30 years since we first wrote a review about its existence; at that time many people believed that with the exception of macropinocytosis, which will only be briefly mentioned in this review, all uptake could be accounted for by clathrin-dependent endocytosis. Now it is generally accepted that there are different clathrin-independent mechanisms, some of them regulated by ligands and membrane lipid composition. They can be both dynamin-dependent and -independent, meaning that the uptake cannot be accounted for by caveolae and other dynamin-dependent processes such as tubular structures that can be induced by toxins, e.g. Shiga toxin, or the fast endophilin mediated endocytosis recently described. Caveolae seem to be mostly quite stable structures with other functions than endocytosis, but evidence suggests that they may have cell-type dependent functions. Although several groups have been working on endocytic mechanisms for years, and new advanced methods have improved our ability to study mechanistic details, there are still a number of important questions we need to address, such as: How many endocytic mechanisms does a cell have? How quantitatively important are they? What about the complexity in polarized cells where clathrin-independent endocytosis is differentially regulated on the apical and basolateral poles? These questions are not easy to answer since one and the same molecule may contribute to more than one process, and manipulating one mechanism can affect another. Also, several inhibitors of endocytic processes commonly used turn out to be less specific than originally thought. We will here describe the current view of clathrin-independent endocytic processes and the challenges in studying them.

## Introduction

Clathrin-dependent endocytosis is still the most studied endocytic pathway; for review, see Kaksonen and Roux (2018), and with the clear basket formed by clathrin, it is perhaps not surprising that the question was asked: how can an endocytic vesicle form without clathrin? Also, published data suggested that all uptake could be accounted for by clathrin-dependent endocytosis (Doxsey et al. 1987). Actually, even a quite recent paper indicated that this can be the case (Bitsikas et al. 2014). However, early suggestions for other processes came from the observation of cholera toxin in caveolae (Montesano et al. 1982), and from the findings that when clathrin was removed by potassium depletion of cells (Moya et al. 1985) or the pinching off of clathrin-coated vesicles was prevented by low cytosolic pH (Sandvig et al. 1987), the protein toxin ricin was still endocytosed. At the time, the counter-arguments raised against clathrin-independent uptake were for instance: when clathrin is removed from the plasma membrane, other elements of the endocytic machinery are still left and may account for a continued uptake. Similarly, when the cytosol is acidified and clathrin-dependent endocytosis is blocked (Sandvig et al. 1987), this might induce compensatory mechanisms that are normally not present. The last argument was used also in the case of removal of clathrin by potassium depletion. However, although newer studies actually revealed that blocking clathrin-dependent uptake can induce increase in clathrin-independent endocytosis (Damke et al. 1995), the finding that both potassium depletion and acidification of the cytosol inhibited uptake of ricin by about 50% fits nicely with the large capacity of the Cdc42-dependent pathway (Howes et al. 2010b). When it comes to the question of the role of caveolae in endocytosis, this has been debated for decades (Hommelgaard et al. 2005; Sandvig et al. 2008; van Deurs et al. 1993; Lamaze et al. 2017; Cheng et al. 2010; Sharma et al. 2004), and a more detailed discussion of caveolae and their role in endocytosis can be found below. It was in fact not until it was published that the dynamin mutant K44A inhibited clathrin-dependent endocytosis without blocking fluid phase uptake that it was generally accepted that there are different endocytic mechanisms (Damke et al. 1994).

It should, however, be remembered that the cellular context is definitely playing a role for quantitative aspect of endocytic uptake. It is an old observation that endocytosis is affected by cell density (Kaplan 1976; Sandvig 1978; Snijder et al. 2009), and increased cell density is associated with changes in lipid composition and intracellular transport (Kavaliauskiene et al. 2014; Frechin et al. 2015). Furthermore, when a cell becomes polarized the clathrin-independent uptake on the two poles are regulated in different ways. The apical clathrin-independent uptake of ricin in polarized MDCK cells can be selectively regulated by protein kinase A, protein kinase C, cyclooxygenase, and by inhibition of calmodulin without any effect on the basolateral uptake (Sandvig and van Deurs 2005). Notably, caveolae are only found on the basolateral pole in MDCK cells (Vogel et al. 1998; Scheiffele et al. 1998).

**Fig. 1:**
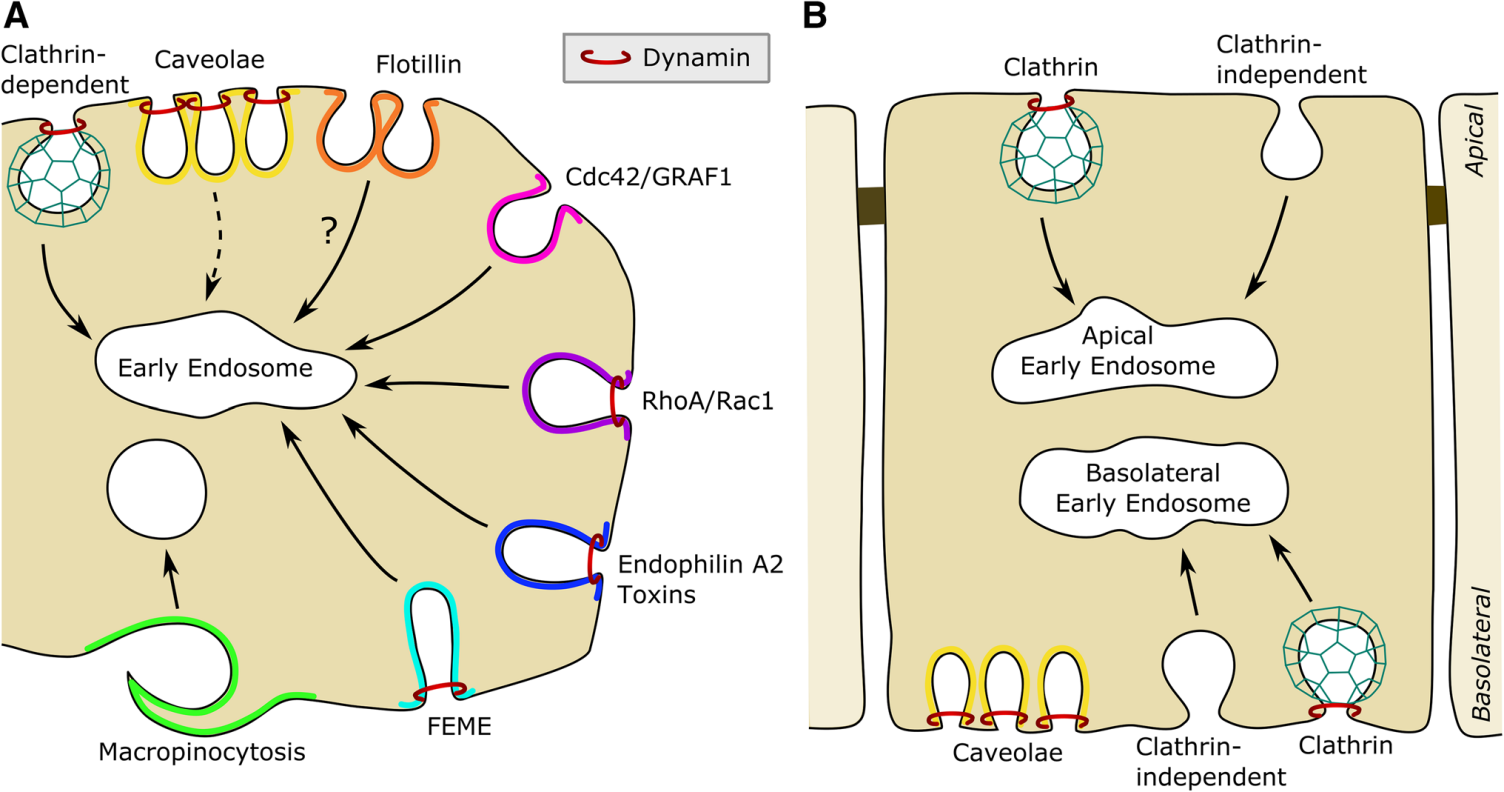
An overview of endocytic mechanisms in **a** non-polarized and **b** polarized epithelial cells. The different mechanisms are described in the text: In addition to clathrin-dependent endocytosis, we have indicated the following pathways: Caveolae, now regarded as quite stable structures; flotillin, may mediate transfer of a ligand to the invagination rather than contribute to the endocytic process; the Cdc42- dependent but dynamin-independent uptake; the Rho/Rac pathway; the toxin-induced tubules with endophilin; FEME (see text); as well as macropinocytosis. In polarized cells the apical clathrin-independent uptake is regulated by a number of factors, e.g. protein kinase A, which does not affect the basolateral uptake. It should be noted that in MDCK cells all caveolae are at the basolateral pole

Although molecular biology techniques have provided us with new possibilities to manipulate and study cellular mechanisms, we will in this article also touch upon the pitfalls and challenges in studies of endocytosis. For instance, overexpression of proteins or mutant proteins may cause unexpected phenotypes; one may sequester proteins due to high concentrations of partners with a low affinity, and overexpression or treatment with siRNA may cause compensatory mechanisms; an example being the upregulation of Rab6A’ upon knockdown of Rab6A (Utskarpen et al. 2006). Importantly, it is also essential in such studies with a strict quantification of what is really internalized and what is still connected to the surrounding of the cell, but present in invaginations that have not pinched off. An overview of endocytic pathways covered in this review can be found in Fig. 1.

### Quantitative aspects of endocytic pathways

**Fig. 2:**
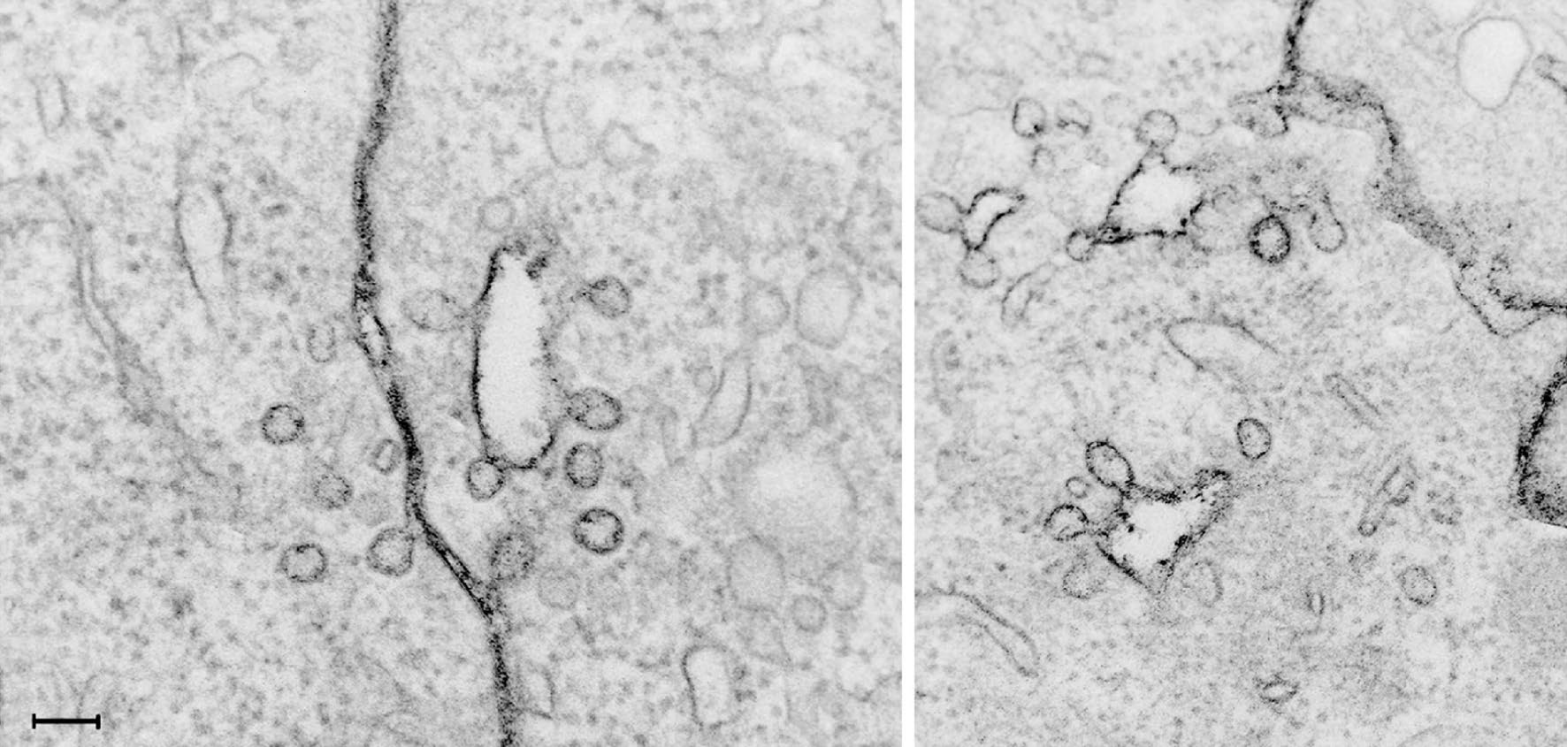
Ruthenium red added during fixation of cells and the resulting black staining of the membrane reveal that caveolae which may appear as free vesicles in the cytosol are surface connected. *Bar* 100 nm. This figure is reproduced from (Sandvig et al. 2011). 10.1016/j.ceb.2011.03.007

There are several challenges when one wants to study quantitative aspects of different endocytic mechanisms, and to answer the question: by which mechanism is a given ligand taken into cells? An important aspect in studies of endocytic uptake of plasma membrane and ligands is: how can one make sure that the ligand under investigation is in a closed vesicle and not only in a deeply invaginated portion of the plasma membrane? When cells are fixed and studied by EM one cannot without serial sectioning conclude as to whether a structure seen still might be connected to the cell surface. An alternative to serial sectioning is fixation in the presence of Ruthenium red which can reveal whether a structure is surface connected (Sandvig et al. 2008, 2011; Caldieri et al. 2017; Liberali et al. 2008). As seen in Fig. 2 caveolae which are still connected to the cell surface can appear to be located far from the cell surface unless the membrane connection is revealed by Ruthenium red staining. Another way to study whether a ligand is internalized is to use high-resolution confocal microscopy with Z-stacks (Iversen et al. 2011). Furthermore, if one wants to study a possible constitutive endocytic process, it is of course important to investigate whether the ligands or compounds added do not in themselves modulate the process. For instance, it was discovered that addition of glycosphingolipids may increase the pinching off of caveolae (Sharma et al. 2004), and to our knowledge it has not yet been investigated to which extent cholera toxin, which can be seen localized in caveolae (Montesano et al. 1982) and which will crosslink the glycosphingolipid GM1, might affect the pinching off of caveolae. Interesting in this connections is that crosslinking of GM1 with the cholera toxin B pentamer can induce transmembrane signaling (Schnitzler et al. 2007) and increase the cytosolic concentration of Ca^2+^ (Ledeen and Wu 2015; Dixon et al. 1987).

Another challenge it is that one and the same protein can be involved in more than one endocytic pathway, an example being endophilin A2 which plays a role both for clathrin-dependent and clathrin-independent uptake (Boucrot et al. 2015; Ferreira and Boucrot 2018; Renard et al. 2015; Hohendahl et al. 2017). Thus, modifying the level or transfecting with mutants are likely to affect different mechanisms. It should be noted that endophilin was recently reported to inhibit dynamin-mediated membrane fission (Hohendahl et al. 2017). However, since a fast endophilin A2-dependent endocytosis (FEME) pathway was reported, it is possible that endophilin can facilitate dynamin-dependent uptake in certain contexts (Boucrot et al. 2015).

Similarly, Cdc42 is important for both macropinocytosis, FEME as well as for the dynamin-independent CLIC/GEEC (clathrin-independent carrier/glycosylphosphatidylinositol (GPI)-anchored protein-enriched endosomal compartments) pathway (Howes et al. 2010a; Sabharanjak et al. 2002; Ferreira and Boucrot 2018). However, inhibition of Cdc42 may increase FEME (Boucrot et al. 2015). Already in 2010 it was reported that phosphocaveolin-1 is involved in the coregulation of caveolar and Cdc42-dependent fluid phase uptake (Cheng et al. 2010), and it turns out that also the caveolar protein cavin can down-regulate the Cdc42 dependent pathway (Chaudhary et al. 2014).

The role of actin in clathrin-dependent endocytosis has been debated over the years. Its involvement is not always obligatory or easy to demonstrate, and was reported to be dependent on cell type and growth conditions (Fujimoto et al. 2000). More recently, the dependency on actin was found to be regulated by membrane tension (Boulant et al. 2011). In agreement with that, insertion of lysolipids with large headgroups into the plasma membrane of HEp-2 cells reduced the clathrin-dependent uptake of transferrin, and made transferrin uptake more dependent on actin (Ailte et al. 2017). It is possible that actin may facilitate all types of endocytosis, but that the requirement varies, as other factors such as protein crowding and insertion of amphipatic helixes can also be involved in vesicle formation (Renard et al. 2018). Thus, the disruption of actin function cannot easily be used to conclude about the quantitative aspects of endocytic pathways.

A common way to study by which mechanisms a given ligand or compound is taken up is to use inhibitors of different enzymes, or chemical compounds affecting various cellular processes. Such studies have to be performed with care, as there are several pitfalls (Iversen et al. 2011). Although the term “well-established small molecule inhibitors” is often used, the conclusions can still be wrong. For instance, the tyrosine kinase inhibitor genistein is often used as a specific inhibitor for uptake by caveolae, but since it is a general inhibitor of phosphotyrosine kinases, it will also inhibit other processes dependent on tyrosine phosphorylation, as for instance uptake of EGF by clathrin-coated pits (Yang et al. 1996) and ruffling induced by growth factors acting via tyrosine phosphorylation (Jiang et al. 1995). So, when the conclusion is that rather large particles (several hundred nanometers) enter via the small caveolae (about 80 nm diameter) since the uptake is inhibited by genistein (Wolfram et al. 2017), one should question whether another mechanism is involved, and it is absolutely worthwhile to recheck what is going on in this system. Another commonly misused approach is to conclude about the involvement of caveolae from studies involving extraction of cholesterol with cyclodextrin. Removal of cholesterol will affect several endocytic pathways, and this is further discussed below. Actually, a number of compounds commonly used to interfere with various endocytic pathways were recently found to affect endophilin A2-positive assemblies (Boucrot et al. 2015), but whether this correlates with inhibition of endocytosis does not seem to be clear.

One should also be aware of the fact that crosslinking of surface structures might induce endocytic uptake in itself. An example is that particles with several Tat peptides can induce recruitment of Rac1 and induce macropinocytosis (Imamura et al. 2011). Also, particles with the plant toxin ricin immobilized on the surface can, in contrast to free ricin, induce a macropinocytotic process (Iversen et al. 2012). Moreover, as suggested by Ferreira and Boucrot (2018), results reported for the uptake of IL-2 (interleukin 2) receptor beta subunit might be affected by use of antibodies against this subunit.

### Membrane lipids and clathrin-independent endocytosis

**Fig. 3:**
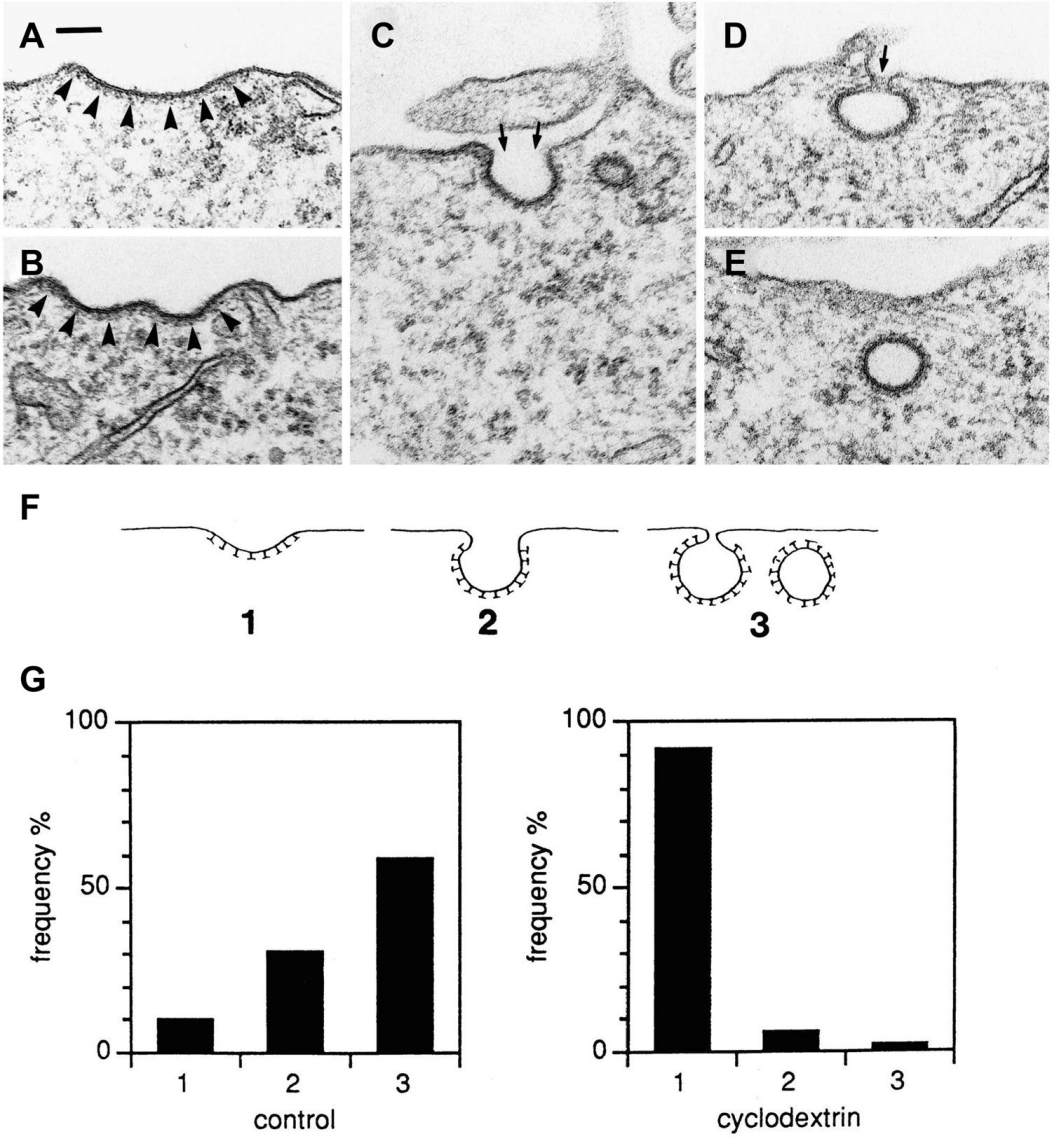
Examples from HEp-2 cells of clathrin-coated areas with different extent of invagination (**a**–**e**) and schematically drawn in **f**. In **g**, clathrin-coats in control cells and cells treated with 10 mM mβCD for 15 min were scored for invagination as classified in **f** (approximately 200 coated pits in each experiment). The relative frequency of the different types is shown, and reveal that mβCD prevents invagination of clathrin-coated pits. Bar 100 nm. This figure is reproduced from (Rodal et al. 1999). http://www.molbiolcell.org/content/10/4/961.full.pdf+html

**Fig. 4:**
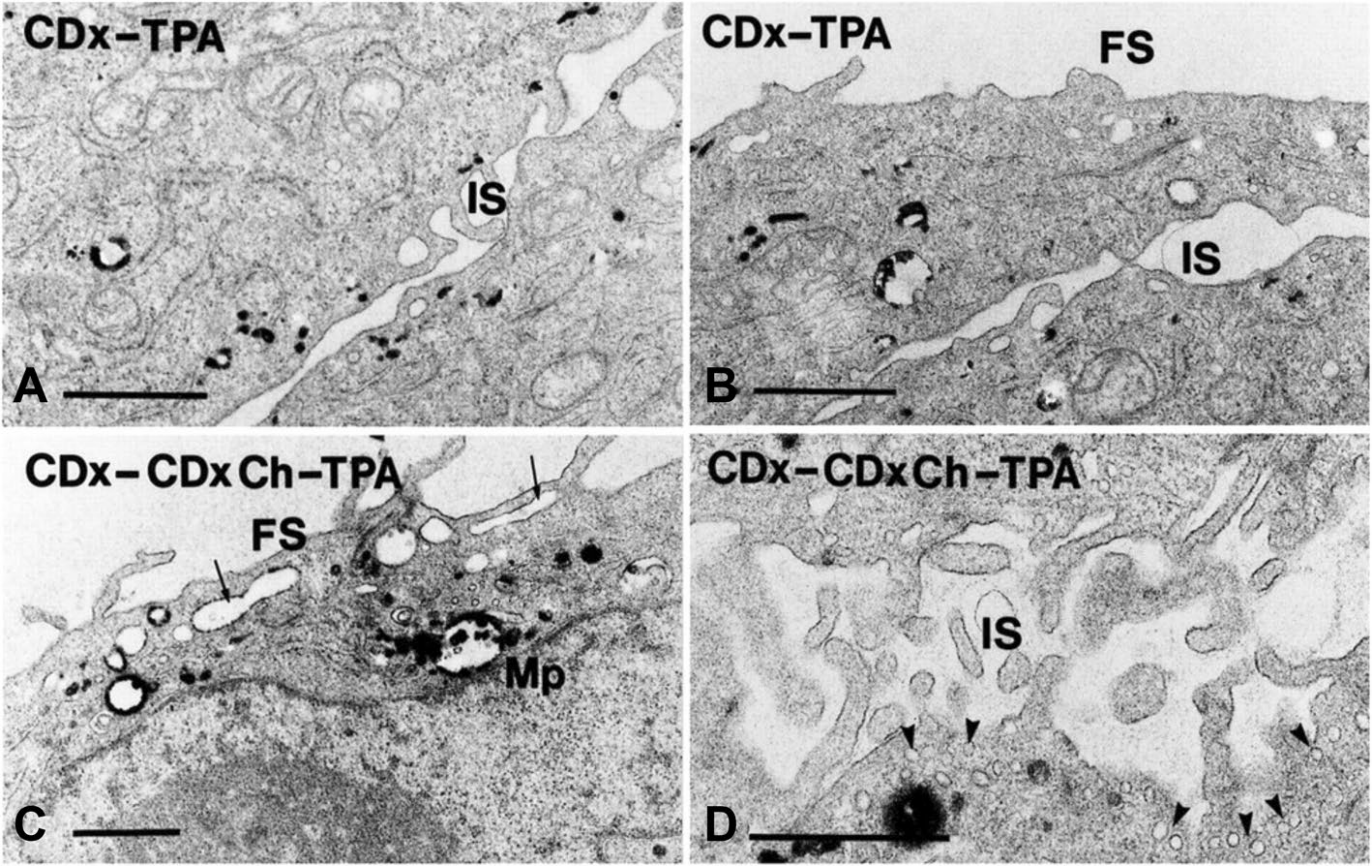
Cholesterol is required for macropinocytosis. A431 cells were serum-starved for 4 h, and in **a** and **b** the cholesterol was lowered by a 30 min incubation with 5 mM mβCD (CDx) before the cells were incubate for 15 min with HRP (horseradish peroxidase) (10 mg/ml) in the presence of mβCD and 1 µM TPA (12-*O*-tetradecanoylphorbol 13-acetate). There is no ruffling and macropinocytosis. In contrast, in **c** and **d**, a mixture of mβCD (2.5 mM) and mβCD (2.5 mM) with cholesterol (CDxCh) was used in order not to affect membrane cholesterol, and as shown, the cells are then able to form ruffles and macropinosomes. Bars 1 µm; *FS* free surface; *IS* intercellular space. This figure was reproduced from (Grimmer et al. 2002); http://jcs.biologists.org/content/joces/115/14/2953.full.pdf

It has been known for many years that the structure of caveolae is dependent on cholesterol, and that addition of cholesterol-binding compounds such as nystatin and filipin, or removal of cholesterol by using methyl β-cyclodextrin (mβCD) will remove the caveolar structures (Rothberg et al. 1990; Klein et al. 1995). However, also clathrin-dependent endocytosis can be reduced by extraction of cholesterol, leaving flat clathrin-coated areas at the plasma membrane (Fig. 3) (Rodal et al. 1999; Subtil et al. 1999). The mechanism behind this change is not understood. Importantly, endocytosis can still occur under these conditions (Rodal et al. 1999). By testing the ability of different sterols to restore endocytosis, it was recently shown that the 3β-OH group in cholesterol is essential and so is the ability of the sterol to support lipid raft formation (Kim et al. 2017). However, it could be that it is the effect of lipid packing and not raft formation that is important (Kim et al. 2017). Also macropinocytosis requires cholesterol; reduction of cholesterol will inhibit recruitment of Rac and the membrane ruffling (Fig. 4) which is a prerequisite for macropinocytosis (Grimmer et al. 2002). Moreover, cholesterol has been shown to be important both for Cdc42-dependent endocytosis (Chadda et al. 2007) and for RhoA-dependent endocytosis of IL-2 receptors (Lamaze et al. 2001). Whether cholesterol redistribution might be involved in the shear stress induced Cdc42-activated apical endocytosis in proximal tubule cells, a process regulated by activators and inhibitors of calmodulin (Bhattacharyya et al. 2017), has so far not been investigated. It should be noted that also clathrin-independent endocytosis on the apical pole of polarized MDCK cells is regulated by calmodulin (Llorente et al. 1996).

In contrast to the examples above, where cholesterol is required for uptake, we found that when clathrin-dependent uptake is blocked by expression of clathrin heavy chain antisense RNA, Shiga toxin uptake after cholesterol depletion is actually increased, suggesting that rafts may prevent endocytosis and keep ligands at the cell surface (Sandvig et al. 2008). Similarly, the raft-associated proteins caveolin and flotillin may actually inhibit uptake from the cell surface of certain ligands. Caveolin-1 was found to inhibit dynamin-dependent, raft-mediated endocytosis of cholera toxin (Lajoie et al. 2009), and flotillin stabilized the plasma membrane association of ErbB receptors (Asp et al. 2014; Pust et al. 2013). After knockdown of flotillin, the ErbB receptors were internalized.

Recent data have revealed that removal of cholesterol also induces formation of tubular structures (Shen et al. 2014; Hirama et al. 2017) that may pinch off in an ATP-independent manner. This is presumably linked to increased charge density at the inner leaflet caused by phosphatidylserine (PS), and similar tubular structures can be created by direct addition of PS (Hirama et al. 2017). In agreement with these findings are data published already in 1995, that an increase in plasma membrane PS would increase endocytosis (Farge 1995). Thus, changing the membrane lipid composition may throw light on the processes normally occurring, but may also create phenomena normally not observed.

Not surprisingly, interfering with sphingolipid synthesis may affect clathrin-independent endocytosis (Cheng et al. 2006). Sphingomyelin was reported to be important for membrane recruitment of both Cdc42 and RhoA, and as mentioned above, addition of certain lipids such as the glycosphingolipid LacCer (lactosylceramide) can induce pinching off of caveolae (Sharma et al. 2004). Thus, trying to follow an endocytic process by inserting probes into the membrane might change the process under investigation. Moreover, changing the membrane fluidity by incubating cells with unsaturated fatty acids was found to reduce the uptake of Shiga toxin, which is internalized by both clathrin-dependent and -independent endocytosis (Spilsberg et al. 2007). Interestingly, crosslinking of glycolipids by ligands such as Shiga toxin and cholera toxin has been reported to induce formation of tubular structures that pinch of in a dynamin- and endophilin-dependent manner (see below) (Römer et al. 2007; Renard et al. 2015).

Changing membrane structure by modifying lipids with enzymes, thereby changing their shape, do also affect the membrane curvature and endocytic uptake. For instance, a wounded cell may, due to a membrane damage causing leakage, get a higher cytosolic Ca^2+^-level, which may induce fusion of lysosomes with the plasma membrane and release of sphingomyelinase with subsequent formation of ceramide from sphingomyelin. Due to the decrease in lipid head group size in the outer leaflet of the plasma membrane, this will facilitate membrane bending and endocytosis of the lesion (Tam et al. 2010). In contrast, insertion of lysophosphatidylinositol with large headgroups into the plasma membrane made it easier to pull the membrane outwards with optical tweezers, in agreement with the idea that it created a positive membrane curvature and made it more difficult to form endocytic structures. Such treatment efficiently reduced clathrin-dependent uptake, whereas it had a much smaller effect on clathrin-independent uptake (Ailte et al. 2017).

To investigate the mechanisms of endocytosis, one may use planar lipid model membranes or liposomes. However, mostly the membrane composition of these membranes does not reflect what is normally found in the plasma membrane. Such model membranes are normally symmetric and contain only a few lipid species, often present in very low amounts in cells. Furthemore, two model systems may not always give the same results. For instance, using Shiga toxin to induce tubules after binding to Gb3, it was found that the semisynthetic C22:1 Gb3, but not C22:0, behaved like porcine kidney Gb3 in the liposome model (Römer et al. 2007). However, in planar lipids C22:0 Gb3 gave similar results as the Gb3 mixture from porcine kidney (Windschiegl et al. 2009), revealing a model-dependent difference that so far is not explained.

### Clathrin-independent and dynamin-dependent endocytic mechanisms

#### RhoA, FEME, Shiga toxin-induced tubules, and ARF6

Clathrin-independent endocytosis can be both dynamin dependent and dynamin independent. Mechanisms dependent on dynamin for vesicle formation are the RhoA-dependent pathway responsible for uptake of IL-2, pinching off of tubules induced by crosslinking of glycolipids by multivalent toxins such as Shiga toxins, the relatively newly described process FEME, and also endocytosis of the EGFR (epidermal growth factor receptor) occurring at high concentrations of EGF (epidermal growth factor). Furthermore, uptake from caveolae and closure of macropinocytic vesicles originating from circular ruffles also require dynamin. However, more than one mechanism may be involved in vesicle formation (Renard et al. 2018). The different dynamin-dependent mechanisms are described in detail below.

In 2015 it was described that pinching off of Shiga toxin induced tubules is dependent not only on dynamin, but also on endophilin and actin (Renard et al. 2015). For a recent review of this mechanism, see Johannes (2017). It should, however, be noted that although endophilin A2 and dynamin knockdown protected cells against Shiga toxin by a factor of 2–3, very little protection was obtained after disruption of actin (Renard et al. 2015), a finding that may be related to Shiga toxin taken in by other mechanisms; both clathrin-dependent and dynamin-independent endocytosis of the toxin occurs in several cell lines; for review, see Bergan et al. (2012) and Sandvig et al. (2014). Furthermore, one should be aware of that dynamin is important also for endosome to Golgi transport of Shiga toxin (Lauvrak et al. 2004; Saint-Pol et al. 2004), thus a protection obtained by knockdown of dynamin is not necessarily related only to uptake from the cell surface. It would certainly be interesting to know the fraction of Shiga toxin taken in by such tubular structures in different cell lines as clathrin-dependent and dynamin-independent uptake of this toxin can account for a considerable uptake of this toxin; for review, see Sandvig et al. (2014). Another study showing the importance of endophilin A2 in clathrin-independent endocytosis was published at the same time (Boucrot et al. 2015). An endocytic pathway operating at the leading edges of cells was demonstrated, and it was found to be ligand triggered and responsible for uptake of a number of different receptors, including the IL-2 receptor. This process, FEME, was reported to be activated upon inhibition of Cdc42, and remarkably seemed to be affected by a large number of inhibitors normally used to interfere with other endocytic pathways.

The RhoA and dynamin-dependent uptake of IL-2 receptor subunits was first described in 2001 (Lamaze et al. 2001), and today several molecules required for this process are known (Basquin et al. 2013). This includes Rac1, Pak and phosphatidylinositol 3-kinase (PI3K), so during the years the mechanistic insight has increased, and the requirements for uptake seem to fit with the recently described FEME mechanism (Boucrot et al. 2015). However, although IL-2 was reported to be internalized by FEME in T cells (Boucrot et al. 2015), it has been suggested that the characteristics found for uptake of IL-2 receptor subunits after crosslinking with antibodies are affected by the antibodies, and that the receptor subunits in spite of many similarities are endocytosed by a different mechanism than FEME (Ferreira and Boucrot 2018). A recent publication reports that endophilin A2 is required for dynamin recruitment during endocytosis of the IL-2 receptor (Bertot et al. 2018). In contrast, dynamin was still recruited to clathrin-coated pits after knockdown of endophilin A2, and importantly, in the control situation endophilin was recruited to all clathrin-coated pits, not only to a subpopulation, meaning that endophilin cannot be used as a marker for clathrin-independent endocytosis. Concerning involvement of Rho proteins in endocytosis of other ligands, it was reported that in basolaterally permeabilized MDCK cells, apical clathrin-independent endocytosis of the plant toxin ricin was inhibited by the *Clostridium botulinum* C3 transferase, implicating involvement of Rho-proteins (Garred et al. 2001). Clearly more studies should be performed to characterize regulation of clathrin-independent uptake in polarized cells.

Also the small GTP-binding protein Arf6 has been implicated in endocytosis; for review, see Grant and Donaldson (2009). However, although this protein was reported to be involved in clathrin-independent, but dynamin-dependent endocytosis of Coxsackievirus A9 (Heikkila et al. 2010) and the Herpes Simplex virus protein VP22 (Nishi and Saigo 2007), Arf6 seems to mainly play a role in recycling (Grant and Donaldson 2009).

Also β1-integrin can be endocytosed by clathrin-independent and dynamin-dependent endocytosis, and tubules that pinch off can be induced by the calmodulin-inhibitor W7 (Soriano-Castell et al. 2017). Interestingly this tubulation was counteracted by active Rac, phospholipase C, the Rac effector ROCK1, and actomyosin at the cell cortex. Similarly, apical clathrin-independent endocytosis in polarized MDCK cells was stimulated by the calmodulin inhibitor W7, whereas there was no effect on the basolateral side (Llorente et al. 1996). However, in this case it was not investigated whether the increased uptake is dynamin dependent.

#### Caveolae/caveolin and endocytosis

Caveolae have been reported to be important for uptake of albumin in endothelial cells (Schnitzer et al. 1994), and the possibility exists that there are different types of caveolae with specialized functions as for instance dynamin-2 is not found in all caveolae (Lamaze et al. 2017). Caveolae are today, at least in most cells, considered as quite stable membrane structures (van Deurs et al. 2003; Pelkmans et al. 2004; Parton et al. 2006; Kirkham et al. 2005) with a role in signaling and in providing membrane during stretching of the plasma membrane (Bastiani and Parton 2010). Thus the observation of a certain ligand in caveolae does not mean that this is the main endocytic pathway for that ligand. An example is cholera toxin which many years ago was found to localize in caveolae (Montesano et al. 1982), and which for years was believed to enter mainly by this pathway. However, quantitative measurements revealed that the presence or absence of caveolae may not play any difference for the uptake of this toxin in Caco-2 cells (Torgersen et al. 2001), and the toxin can be endocytosed also by clathrin-dependent endocytosis (Torgersen et al. 2001; Shogomori and Futerman 2001; Nichols et al. 2001). At the time when these data were published this caused some reaction, but this view is now accepted as different groups published similar results. As mentioned above, certain ligands and lipids may induce pinching off of caveolae, and it would be interesting to know if cholera toxin-induced cross-linking of GM1 actually might induce pinching off of caveolae. Relevant in this connection is the finding that the virus SV40, which also binds multivalently to glycolipids, can enter through caveolae (Pelkmans et al. 2001), but that it may enter even more efficiently in cells without these structures (Damm et al. 2005). Also, it should be noted that at a time it was believed that SV40 went from caveolae to the so-called neutral “caveosome” and from there to the ER, and actually it is still being published that people try to target for instance nanoparticles to these structures to avoid the endosomal/lysosomal pathway and degradation of the particles. However, it is important to be aware of that the caveosome turned out to be an artefact, and that the content of caveolae after pinching off ends up in the endosomal pathway (Parton and Howes 2010). Actually the authors of the original articles about the caveosome have suggested that the term “caveosome” should not be used any more (Hayer et al. 2010).

Caveolin is found not only in caveolae, but also on flat areas of the plasma membrane where it may function to restrict and not facilitate uptake of certain ligands. This has been reported to be the case for uptake of autocrine mobility factor (AMF) and for cholera toxin (Lajoie and Nabi 2010). A recently published mechanism involving caveolae, but apparently not their endocytic function, concerns their involvement in an ARF6-dependent mechanism linking hemidesmosome remodeling and mechanoresponse (Osmani et al. 2018).

#### EGFR-reticulon 3 dependent uptake

It has been known for some time that at high concentrations of EGF, the EGF/EGFR complex is endocytosed by a clathrin-independent mechanism leading to degradation of the growth factor, and recently it was described that EGF is able to induce increased contact between the plasma membrane and the ER (endoplasmic reticulum) in a reticulon 3 dependent mechanism. An IP3 (inositoltrisphosphate)-dependent release of Ca^2+^ from the ER is required for the uptake of EGFR, and in addition dynamin is involved. The plasma membrane protein CD147 was found to be cointernalized with EGFR. Whether other growth factors or ligands may be endocytosed by a similar mechanism, is still an open question.

### Dynamin-independent endocytosis

#### Cdc42 dependent endocytosis giving rise to small vesicles

The Cdc42 dependent pathway, also called CLIC/GEEC pathway (Howes et al. 2010a; Sabharanjak et al. 2002; Ferreira and Boucrot 2018), is responsible for quite a fraction of fluid phase uptake in cells, and it has been reported to take in GPI-anchored proteins, protein toxins that bind glycolipids as well as transmembrane proteins such as CD44; for review see Bendris and Schmid (2017). Perhaps a bit surprising, this Cdc42-dependent form of endocytosis is dynamin independent even though one of the proteins implicated in the process, GRAF1, is able to bind dynamin (Ferreira and Boucrot 2018). However, in this context GRAF1 seems to function as a GAP for Cdc42 (Bendris and Schmid 2017). Furthermore, SNX9 is a positive regulator of Cdc42, and knockdown of SNX9 reduces the uptake of CD44 (Bendris and Schmid 2017). Interestingly, this sorting nexin is involved also in clathrin-dependent endocytosis and may serve as a negative regulator of RhoA; for a recent review on the role of SNX9 in endocytosis, see Bendris and Schmid (2017).

ARF1 and its GEF, GBF1, are also reported to be important for this Cdc42-dependent clathrin-independent uptake; for review, see Kaczmarek et al. (2017). Remarkably, although CtBP/BARS seems to be important for fluid phase uptake, a direct involvement in the Cdc42- dependent pathway described above has to our knowledge not been demonstrated. On the other hand, it has been demonstrated to be involved in uptake by macropinocytosis (see below).

#### CtBP/BARS and associated proteins

CtBP/BARS was found to be important both for fission at the plasma membrane and in the Golgi apparatus, and interfering with its function in COS7 cells reduced fluid phase uptake by 60% (Bonazzi et al. 2005). However, although the mechanistic details are still not clarified, different proteins associated with CtBP/BARS as well as its state of phosphorylation are important for its function (Liberali et al. 2008; Valente et al. 2013). It has been suggested that the protein 14-3-3γ binds and stabilizes the phosphorylated fission active monomeric form of CtBP/BARS in connection with macropinocytosis. This does not exclude involvement of other partners. Interestingly, ARF1, which is also involved in macropinocytosis, stimulates together with CtBP/BARS the enzyme PLD (phospholipase D) in a synergic manner. The locally generated PA (phosphatidic acid) is important for the fission; for review, see Valente et al. (2013). To which extent CtBP/BARS is involved in different endocytosis mechanisms does not seem to be known, and several fission mechanisms may play a role in the different endocytic processes; for review, see Renard et al. (2018).

#### Macropinocytosis

In this review, we will not describe macropinocytosis in detail since it differs from the other endocytic mechanisms by resulting in formation of relatively large vesicles, and macropinocytosis has been recognized as an important uptake mechanism for fluid and membrane receptors for many years. We want to refer the reader to some recent reviews (Orth and McNiven 2006; Itoh and Hasegawa 2013; Ferreira and Boucrot 2018; Marques et al. 2017; Buckley and King 2017). Briefly, there are two main types of macropinocytosis: one type involving circular ruffles and being dependent on dynamin for pinching off (Orth and McNiven 2006; Itoh and Hasegawa 2013), the other one being dynamin-independent; for recent reviews, see Ferreira and Boucrot (2018) and Marques et al. (2017). As for other endocytic mechanisms, SNX9 has been found to be a central player also for macropinocytosis (Bendris and Schmid 2017). As mentioned above, CtBP/BARS is involved in macropinocytosis, and it has been reported that completion of macropinocytosis is dependent on the conversion of phosphoinositides (Maekawa et al. 2014). It should be mentioned that a commonly used method to block macropinocytosis is to inhibit the Na^+^/H^+^-exchanger by addition of amiloride or derivatives of this drug, thereby lowering the submembranous pH and inhibiting recruitment of Rac and Cdc42 (Koivusalo et al. 2010). However, these drugs were also reported to affect endophilin-positive assemblies in cells (Boucrot et al. 2015), and in general care should be taken in interpreting inhibitor-based studies.

#### Flotillins and their role in endocytosis

The raft-associated proteins flotillin 1 and 2 have also been implicated in endocytosis (Glebov et al. 2006; Sandvig et al. 2011). Knockdown of flotillin-1 was found to inhibit uptake of GPI-linked proteins (Glebov et al. 2006). However, a very low frequency of flotillin-microdomain associated budding has been reported (Riento et al. 2009), a finding in agreement with the lack of effect of flotillin-depletion on the rate of cholera toxin endocytosis (Saslowsky et al. 2010). Also, there was no effect on the uptake of the plant toxin ricin and uptake of Shiga toxin upon knockdown of flotillin-1 and flotillin-2 (Pust et al. 2010). Interestingly, flotillin microdomains may, in a similar way as reported for flat membrane domains containing caveolin, prevent endocytosis of membrane receptors. We recently reported that ErbB receptor internalization is induced by knockdown of either flotillin-1 or flotillin-2 (Asp et al. 2014). In cases where flotillins actually mediate uptake, it is possible that they are important for pre-endocytic clustering, and not for the endocytic process itself, and the term flotillin-assisted endocytosis (instead of flotillin-dependent endocytosis) has therefore been suggested (Meister and Tikkanen 2014).

## Conclusion

As described in the current review, the scientific community still has a long way to go when it comes to understanding the different endocytic mechanisms; the molecular details, the influence of cell context such as polarization, the effect of ligands with the possibility of crosslinking and mediating transmembrane signaling, as well as the variability between cell types.
